# The Broad Spectrum of Gallbladder Paraneoplastic Syndromes

**DOI:** 10.1016/j.gastha.2023.12.005

**Published:** 2023-12-22

**Authors:** Shuhaib Ali, Mukarram Jamat Ali, Ammad Javaid Chaudhary, Saad Ur Rehman, Muhammad Arqam Maqsood

**Affiliations:** 1 Department of Internal Medicine, University of Texas Health Science Center, San Antonio, Texas; 2 Department of Internal Medicine, Howard University Hospital, Washington, District of Columbia; 3 Department of Internal Medicine, Henry Ford Hospital, Detroit, Michigan; 4 Internal Medicine, Carle Illinois College of Medicine, Urbana, Illinois; 5 Community Medicine, Combined Military Hospital Lahore Medical College, Faisalabad, Pakistan

**Keywords:** Gallbladder Carcinoma, Paraneoplastic Syndromes, Diagnosis, Neoplastic Disease, Treatment Outcomes

## Abstract

Gallbladder carcinoma (GBC) is a rare gastrointestinal tumor with a reported incidence of 1 in 100,000 in the United States. GBC may present with subtle signs and symptoms that can be missed on routine examination and/or confused with other conditions. Unfortunately, its subtle presentation frequently leads to late diagnosis and, thus, a poor prognosis. Several paraneoplastic syndromes have been associated with GBC. Despite their strong associations with neoplastic disease, the precise pathophysiologic mechanisms underlying the development of these syndromes remain poorly understood. Given the vague nature of their initial signs and symptoms, these syndromes are frequently diagnosed as independent entities and only later associated with occult malignancies that may have already metastasized to other organs. Physicians need to be aware of the signs and symptoms of these paraneoplastic syndromes and include an underlying malignancy as part of the differential diagnosis. This review provides a detailed discussion of the paraneoplastic syndromes associated with GBC.

## Introduction and Background

Gallbladder carcinoma (GBC) is a rare neoplasm that is diagnosed most frequently in women, the elderly, and Native Americans.^1^ The overall incidence of GBC in the United States has been estimated at 1–2 cases per 100,000 persons^2^; approximately 3700 cases were diagnosed in 2007–2011 with a mortality rate of approximately 54%.^3^ The overall survival rate of patients with gallbladder malignancy has been reported to be below 5%.^4^ Gallstones are the most common risk factor associated with GBC. Other causes include primary sclerosing cholangitis, ulcerative colitis, liver flukes, chronic *Salmonella enterica* serovar *Typhi,* and *Helicobacter* infection.^5^ Chronic inflammation plays a main role in the development of GBC. Chronic inflammation provides a conducive environment for the development and progression of cancer. Both the major histogenic pathways (lithiasic or nonlithiasiac) that have been recognized for GBC pathogenesis are driven by chronic inflammation.^6^^,^^7^

The initial symptoms of GBC are frequently subtle and may be vague. The most common findings associated with this disease include pruritus, scleral icterus, cachexia, and chronic abdominal discomfort. Most of these symptoms develop in response to the deposition of bile constituents, which ultimately blocks excretion through the cystic duct. Because the gallbladder is hollow and distensible, it can accommodate a relatively large mass before the bile flow becomes obstructed. Once the mass is large enough to elicit symptoms, the tumor has frequently metastasized to adjacent and distant organs via local, lymphatic, or hematogenous spread.^6^^,^^7^ The diagnosis of GBC is usually based on patient history, physical exam, laboratory results, imaging, and biopsy results. Most patients with obstructive symptoms have a poor prognosis and may need extensive surgical procedures or consider palliative care options. While early detection of this tumor before it has spread to adjacent or distant organs may be curable with surgery, most GBC goes unnoticed because of the subtle nature of the earliest clinical symptoms. Interestingly, ∼1% of specimens collected from routine cholecystectomy procedures present evidence of invasive GBC.^6^ Currently, there are no clinical guidelines for routine screening of patients at risk for developing GBC.

**Figure 1:**
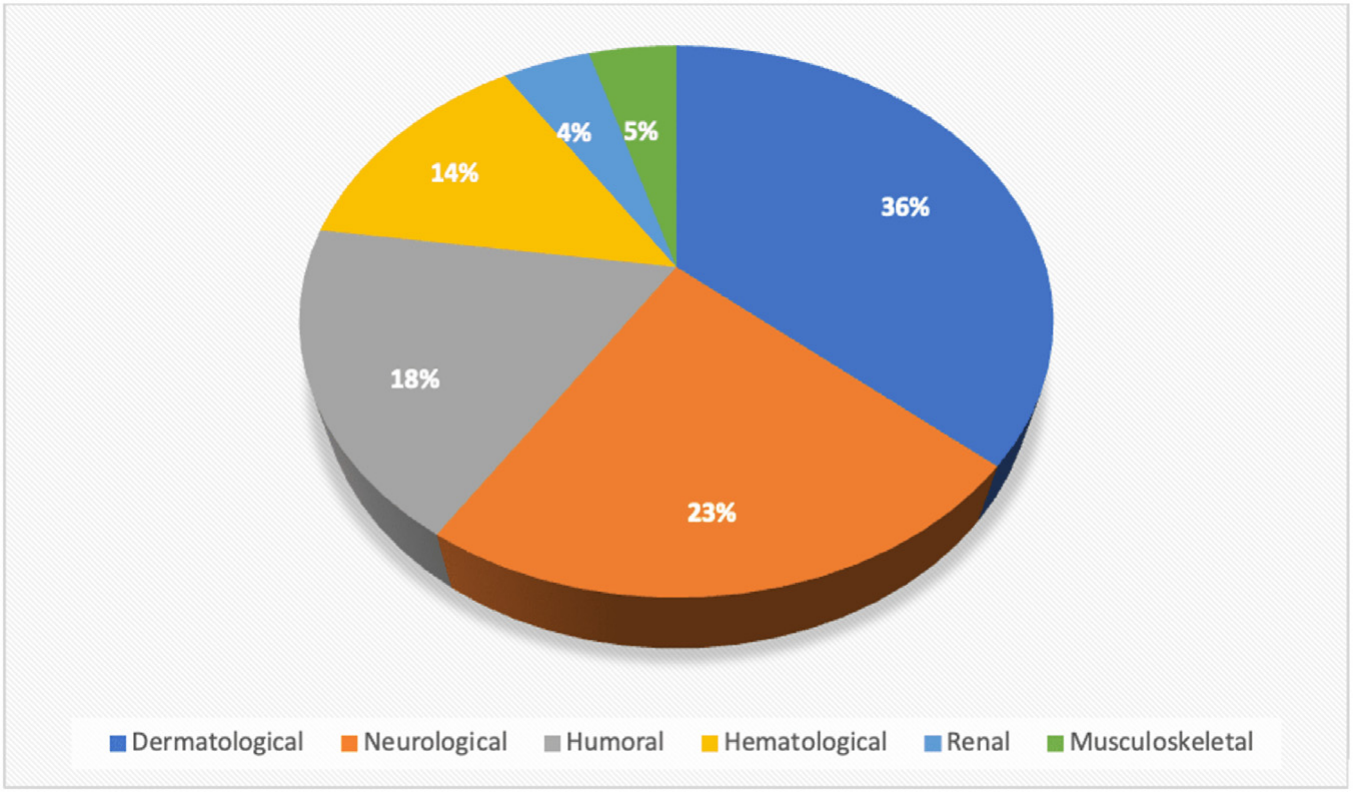
Distribution of paraneoplastic syndromes associated with gallbladder carcinoma.

**Figure 2:**
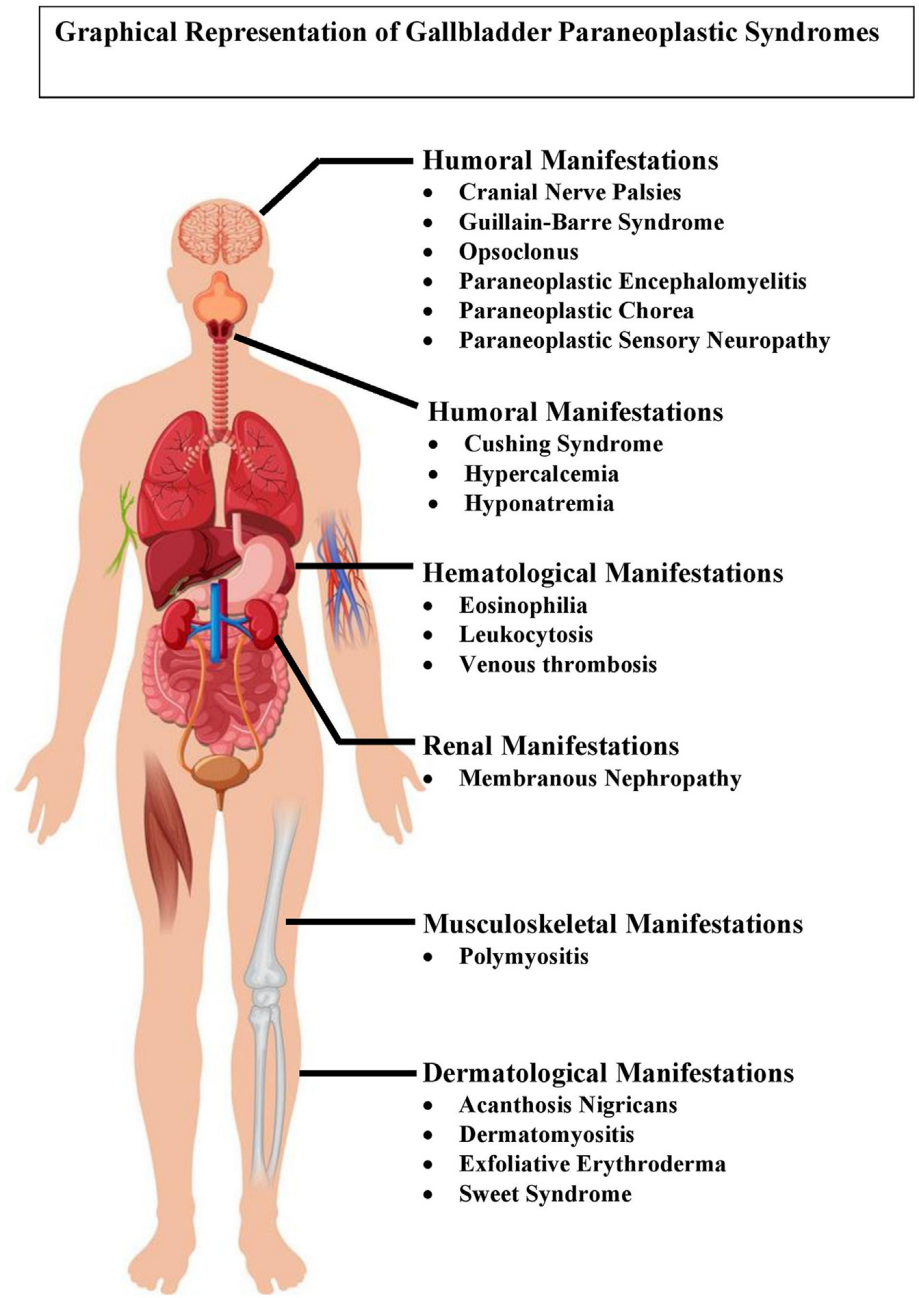
Graphical representation of paraneoplastic syndrome associated with gallbladder cancer.

GBC can also present as a constellation of symptoms that may be attributed to paraneoplastic syndromes (Figures 1 and 2). Paraneoplastic syndromes are a group of clinical disorders typically associated with neoplasms that have no direct effect on the tumor mass or its metastatic lesions.^6^ These syndromes are believed to result from immune-mediated mechanisms,^8^ such as tumor-mediated production of aberrant antigens or mediators that can elicit the characteristic symptoms. While GBC is a rare neoplasm, its primary presentation as a paraneoplastic syndrome occurs even less frequently. Thus, the symptoms associated with this latter disorder may be initially regarded as stand-alone entities (Figure 2).

In this review, we present the results of a literature search of all the reported cases of paraneoplastic syndromes in GBC (Table). These are discussed further in the sections to follow.

**Table:** Summary of Literature on Paraneoplastic Syndromes Associated With GBC

Authors (reference)	Year	Age	Gender	Paraneoplastic syndrome
Dermatologic				
Ramirez^9^	1999	55	F	Acanthosis nigricans
Jurcic^10^	2015	48	F	Dermatomyositis
Yiannopoulos^11^	2002	75	F	Dermatomyositis
Ni^12^	2013	67	F	Dermatomyositis
Narasimhaiah^13^	2011	65	F	Dermatomyositis
Eltawansy^5^	2015	71	M	Exfoliative erythroderma
Kameyama^14^	2005	77	M	Exfoliative erythroderma
Jindal^15^	2012	45	F	Sweet syndrome
Hematological				
Tsunematsu^16^	2019	48	M	Eosinophilia
Nagpal^17^	1998	50	F	Leukocytosis
Wirowski^18^	2008	69	F	Venous thrombosis
Humoral				
Spence^19^	1975	44	F	Cushing’s syndrome
Takahashi^20^	1985	72	F	Hypercalcemia
Ng^21^	2010	35	F	Hyponatremia
Tamura^22^	2013	47	F	Hyponatremia
Musculoskeletal				
Adli^23^	2013	68	F	Polymyositis
Neurological				
Kaido^24^	2016	69	M	Cranial nerve palsies
Phan^25^	1999	70	M	Guillain-Barre syndrome
Corcia^26^	1997	72	F	Opsoclonus
Ogawa^27^	2004	80	F	Paraneoplastic encephalomyelitis
Uribe-Uribe ^8^	2009	48	F	Paraneoplastic sensory neuropathy
Yanai^28^	2021	81	F	Paraneoplastic chorea
Renal				
Hoxha^29^	2016	40	F	Membranous nephropathy

## Review

### Gallbladder Paraneoplastic Syndromes

#### Dermatological manifestations

##### Acanthosis nigricans

Acanthosis nigricans (AN) is one of the most common dermatological paraneoplastic manifestations of cancer, notably those of gastric origin.^30^ To the best of our knowledge, no published studies formally define a link between AN and GBC. Clinically, these lesions are identified on physical examination as velvety hyperpigmented plaques over the forehead and neck with nasal ulcerations. Oral lesions are also detected in 50% of AN cases.^31^ Ramirez et al.^9^ described a patient who presented with oral AN lesions associated with an underlying diagnosis of GBC. On examination of the oral cavity, numerous painless papillomas were detected involving the upper vermilion border, buccal cavity, soft and hard palate, and tongue dorsum. This specific paraneoplastic manifestation has been attributed to the secretion of transforming growth factor-alpha by the neoplastic cells, which leads to the proliferation of keratinocytes in the oral cavity.^32^

##### Dermatomyositis

Dermatomyositis (DM) is an inflammatory myopathy involving the body's proximal muscles and the skin. While this syndrome features several clinical manifestations, it presents most frequently as erythema of the face, neck, and ear accompanied by a butterfly-shaped skin rash and weakness of the proximal muscles.^10^ The results of a study published by Yiannopoulos et al.^11^ described an association linking dysphagia, dysphonia, and eyelid edema with underlying GBC. Similarly, Ni et al.^12^ discussed a case that involved the joints, with symptoms that included arthralgia and morning stiffness in hands for almost 20 days before presentation to a hospital. Narasimhaiah et al.^13^ characterized symptom progression. In this case, proximal muscle weakness and rash emerged 2 months before the patient presented to the hospital; dysphagia and nasal regurgitation ensued an entire month after the initial rash. Symptoms of DM may result from tumor-mediated production and secretion of adrenocorticotropic hormone, growth hormone, and serotonin^12^. While DM is frequently observed in patients with gastric and lung carcinomas, it is rarely associated with GBC.^33^ Only 6 cases of DM associated with GBC were reported from 1976 through 2015. Interestingly, all of these reports featured female patients who were 44 years of age or older. Of note, the time elapsed between detecting the rash and diagnosing carcinoma ranged from 2 weeks to 2 years. Collectively, these findings highlight the need for early screening of patients presenting with signs and symptoms of DM.^10^

##### Exfoliative erythroderma

Exfoliative erythroderma (ED) is another rare paraneoplastic manifestation of GBC. ED presents clinically as a pruritic exfoliating rash widely distributed over the body. Eltawansy et al.^5^ reported a case in which the rash began as a localized lesion; one month later, the rash had spread to the hands, abdomen, knees, and legs. Only 2 cases of ED associated with GBC have been reported in the literature. In addition to the Caucasian patient described by Eltawansy et al.,^5^ the patient reported by Kameyama et al.^14^ was Japanese. While the exact pathophysiologic mechanism via which the tumor triggers ED remains unclear, the resolution of the rash following resection of the primary tumor supports the identification of ED as a paraneoplastic manifestation of GBC.^34^

##### Sweet syndrome

Sweet syndrome (SS), also known as acute febrile neutrophilic dermatosis, is an inflammatory condition that presents with an acute onset of fever and extremely tender, erythematous, fluid-filled vesicles over the back, chest, and arms. The erythematous rash is distributed symmetrically across the body and is associated with ongoing fever, malaise, and nausea.^15^ The lesions associated with paraneoplastic SS, which are bullous, ulcerative, and recurrent,^35^ differ from those associated with the classic form of this syndrome. Paraneoplastic SS may be the result of tumor-associated secretion of cytokines, including interleukin (IL)-1, IL-6, IL-8, granulocyte colony-stimulating factor, and granulocyte monocyte colony-stimulating factor (GM-CSF).^36^ While SS is rarely associated with GBC, treatment of underlying malignancy results in its complete resolution, thereby supporting the association between these 2 conditions.

#### Hematological manifestations

##### Eosinophilia

Eosinophilia is defined as an abnormal increase in eosinophils in the blood and tissues. Tsunematsu et al.^16^ presented the first case of paraneoplastic eosinophilia associated with GBC. Clinically, this patient presented with difficulties with motor function and speech secondary to an underlying Trousseau syndrome, an increase in eosinophils from a baseline level of 5.8% (200 cells/μl) to as high as 14% (3900 cells/μl), and an abnormal thickening of the gallbladder on magnetic resonance cholangiopancreatography.^16^ Several mechanisms have been proposed to explain the prominent eosinophilia. One possibility is that one or more specific proteins released secondary to necrotic changes in carcinoma may trigger an eosinophil chemotactic response. Metastasis of neoplastic cells in the bone marrow may also result in the release of eosinophils. These events may also promote the synthesis and release of eosinophilopoietic factors, including IL-3, IL-5, and GM-CSF, all of which may induce massive eosinophilia by decreasing their rate of apoptosis for as long as 12–14 days.^37^ Eosinophilia may also be linked to disseminated intravascular coagulation, a systemic condition indicative of a poor prognosis.^38^ Prompt diagnosis and management of these issues are imperative in all cases. GBC screening may be considered if other causes of eosinophilia have been ruled out.

##### Leukocytosis

Leukocytosis is defined as an increase in the total number of white blood cells recruited into circulation (>11,000/mm3). Nagpal et al.^17^ described the only known case in which leukocytosis was linked to GBC. This patient presented clinically with anorexia, weight loss, low-grade fever, and total leukocyte counts as high as 66,000, with 95% of polymorphonuclear cells but no evidence of acute infection. The increase in total leukocyte count was attributed to the tumor-associated release of several relevant cytokines, including granulocyte colony-stimulating factor, GM-CSF, IL-1, and IL-3. Paraneoplastic leukocytosis was previously associated with squamous cell carcinomas of the lower jaw, lung, and thyroid, but not with GBC. However, because the symptoms resolved after removing the tumor, we can tentatively attribute this finding to a paraneoplastic syndrome.^39^^,^^40^

##### Venous thrombosis

Venous thrombosis (VT) results from a clot that obstructs the normal outflow of blood from deep veins in the arms, legs, and pelvis. A study by Wirowski et al.^18^ presented a patient case that revealed a paraneoplastic association between complex VT and GBC. Clinically, the patient presented with occlusions of the sigmoid sinus, internal jugular, brachiocephalic, and subclavian veins that failed to respond to oral anticoagulation. Given the grave consequences of refractory VT, it might be prudent to screen these patients for GBC once the common causes of this disorder have been ruled out.

#### Humoral manifestations

##### Cushing syndrome

Cushing syndrome (CS) is a collection of physiological and phenotypic responses to elevated cortisol levels in the plasma. Spence and Burns-Cox^19^ reported the only known case of CS that developed in a patient with GBC. Clinically, CS presents as a gradual increase in body weight, muscle weakness, leg edema, and leukocytosis, as well as an increase in facial hair and impaired cognition secondary to the release of ectopic adrenocorticotropic hormone from an APUDoma of the gallbladder.

##### Hypercalcemia

Hypercalcemia is defined as an increased serum calcium level (>10.5 mg/dl). Takahashi et al.^20^ reported the first case of hypercalcemia as a paraneoplastic syndrome associated with GBC in a patient who presented with right upper quadrant pain, nausea, loss of appetite, weight loss, and leukocytosis. Laboratory studies revealed an elevated serum calcium level of 12.2 mg/dl (normal range, 8–10 mg/dl) that may result from either increased levels of parathyroid hormone or release of colony-stimulating factors from the tumor. In this case, removing the tumor ultimately led to a decrease in the patient’s serum calcium level to near normal (ie, 10.4 mg/dl).

##### Hyponatremia

Hyponatremia is a state in which serum sodium levels have decreased to <135 mEQ/L. While this clinical condition is more commonly associated with small cell carcinoma of the lungs, 2 cases reported in medical literature describe hyponatremia as a complication of underlying GBC. Ng et al.^21^ reported the case of a patient with a 2-week history of epigastric pain radiating to the back, nausea, and vomiting. Laboratory values included a serum sodium level of 107 mEQ/L (normal range, 135–150 mEQ/L) and serum osmolarity of 231 mEQ/L (normal range, 275–300 mEQ/L). A computed tomography (CT) scan revealed a thickening of the wall of the gallbladder. Similarly, Tamura et al.^22^ described a patient who presented with hyponatremia secondary to tumor-associated inappropriate secretion of the antidiuretic hormone as a paraneoplastic manifestation of GBC. Removal of the tumor resulted in substantial (80%) resolution of the symptoms.^41^

#### Musculoskeletal manifestations

##### Polymyositis

Polymyositis is an inflammatory condition that can lead to damage and myopathy of the proximal muscles. While paraneoplastic polymyositis has been reported primarily in cases of ovarian adenocarcinoma,^42^ one published case report links this syndrome to an underlying GBC. Adli et al.^23^ described the clinical presentation of a patient who was ultimately diagnosed with GBC and presented with paraneoplastic polymyositis. The patient reported that these symptoms became significant 2 weeks before hospital admission. A physical examination revealed weakness and tenderness of the proximal muscles with sparing of the distal muscles and a reduction in deep tendon reflexes.

#### Neurological manifestations

##### Cranial nerve palsies

Cranial nerve palsies (CNPs) result in the loss of function of cranial nerves secondary to lesions found anywhere from the central nuclei to the muscles supplied by the nerves. Kaido et al.^24^ described the first known case of paraneoplastic CNP associated with an underlying GBC. Initial symptoms of this condition included double vision and ptosis, leading to complete eye closure within one week. Clinical examination revealed complete paresis of cranial nerves III and VI. While all known potential causes of this condition, including nerve compression, inflammation, infection, diabetes mellitus, and vasculitis, were ruled out, all symptoms resolved within 2 months after surgical removal of a primary GBC.

##### Guillain-Barré syndrome

Guillain-Barré syndrome involves the autoimmune destruction of peripheral nerves by antibodies produced in response to an infectious or neoplastic trigger and is a rare paraneoplastic manifestation. One case of paraneoplastic Guillain-Barré syndrome in association with GBC was reported by Phan et al.^25^ As described in this report, the patient presented with progressive weakness and paresthesias accompanied by decreased reflexes in the upper limbs and absent reflexes in the lower limbs. These symptoms may result from cross-reactions of antitumor antibodies that detect similar epitopes in peripheral nerves.^43^ Hence, GBC screening must be carried out in patients presenting with similar symptoms once all more common causes have been ruled out.

##### Opsoclonus

Opsoclonus is a term used to describe uncontrolled, rapid, involuntary eye movements that may persist even when the eyes are closed. The overall incidence of paraneoplastic opsoclonus is 20%.^44^ This syndrome has been associated with various carcinomas, for example, neuroblastoma,^45^ but has only rarely been detected in patients with GBC. Corcia et al.^26^ reported the first case of opsoclonus linked to an underlying diagnosis of GBC. The symptoms presented in this case included sudden onset of rapid uncontrolled eye movements, followed by impaired consciousness. While all the other causes (ie, infectious, neoplastic, degenerative, or metabolic) had been ruled out, this paraneoplastic syndrome was ultimately linked to the patient’s underlying GBC.

##### Paraneoplastic encephalomyelitis

Paraneoplastic encephalomyelitis (PEM) results from neuronal degeneration secondary to the deposition of lymphocytic infiltrates in and around the central nervous system. Ogawa et al.^27^ presented a case of PEM in a patient with an underlying GBC. The clinical presentation of this syndrome included CNP (primarily facial) and sensory ataxia, together with anorexia and weight loss. Mild muscle weakness developed as the disease progressed, together with loss of the ankle and triceps reflex, sensory loss, and diffuse muscle wasting. Signs and symptoms of PEM may be linked to the aberrant production of anti-Hu antibodies leading to the destruction of large myelinated nerve fibers. Several earlier studies described paraneoplastic motor neuron syndromes secondary to underlying malignancies, but none were explicitly associated with GBC.^46^

##### Paraneoplastic chorea

Paraneoplastic chorea is extremely rare. Small-lung carcinoma is the most common underlying cancer etiology. However, Yanagi et al.^28^ have reported a case of chorea in a patient with underlying GBC. The patient presented with involuntary dance-like movements. Contrast-enhanced magnetic resonance imaging of brain revealed no obvious abnormality. Laboratory tests including autoantibodies were negative except that CA 19-9 levels were markedly elevated. CT abdomen with contrast showed gallbladder cancer with multiple liver metastasis. Chorea was attributed to the underlying GBC as the symptoms satisfied the diagnostic criteria of paraneoplastic neurological syndrome. The symptoms resolved after 2 months of gemcitabine-cisplatin chemotherapy for GBC.

##### Paraneoplastic sensory neuropathy

Paraneoplastic sensory neuropathy (PSN) is a condition associated with loss of sensation due to underlying neoplasia. Neurological signs develop in 80% of patients before the diagnosis of carcinoma of any type is made. While several carcinomas have been associated with PSN, Uribe-Uribe et al.^8^ presented the first case of PSN associated with an underlying GBC. The patient in this study presented with progressive dysesthesia of the upper and lower limbs, followed by anesthesia of the trunk. Several weeks later, the patient reported weakness of the leg muscles, nausea, vomiting, headache, and weight loss.^8^ Similar to PEM, PSN may develop in response to the generation of anti-Hu antibodies that promote the demyelination of peripheral nerve fibers.^47^

#### Renal manifestations

##### Membranous nephropathy

Membranous glomerulonephropathy (MGN) is a type of nephrotic syndrome characterized by the deposition of immune complexes on the walls of the glomeruli leading to thickening and damage of the vessel walls. Results of a study published by Hoxha et al.^29^ described a link between MGN and GBC. Paraneoplastic MGN presents clinically with proteinuria in the nephrotic range (>3.5g/dl), potentially resulting from antibodies that develop against the podocyte antigen, thrombospondin type-1 domain-containing 7A, which has also been detected in neoplastic cells found in the gallbladder.^48^

#### Association of GBC with other neoplasms

Only a few reports document an association or any common mutations shared by GBC with other characterized carcinomas. Although some genetic mutations reported in GBC, for example, *TP53 and KRAS*,^49^ are shared by other neoplasms, no published papers document their mechanistic relationship. Some paraneoplastic syndromes, for example, DM, are detected more frequently in patients diagnosed with other neoplasms, such as lung and colon cancer. However, the current literature provides no clue as to whether diagnosing GBC with DM warrants a workup for lung or colon cancer or vice versa.

#### Early detection of GBC

GBC is currently one of the most lethal neoplasms with an overall survival rate [DE2] that approaches 5%. Thus, methods and protocols that might be used for early detection of GBC remain among the most critical goals for physicians practicing gastroenterology.^4^ At present, there are no clear guidelines regarding the early detection of GBC. Even in high-risk patients (for example, elderly patients with a long history of gallstones or chronic cholecystitis), a postoperative histological examination is the only reliable diagnostic tool available. Using ultrasound imaging methods remains limited, as it can be difficult to distinguish between a neoplasm and gallstones. Preoperative biopsies are invasive and lead to significant concerns regarding tumor seeding.

Similarly, while CT/magnetic resonance imaging scans can help diagnose GBC, they are not ordered routinely in patients who present with the symptoms of cholecystitis or cholelithiasis; this increases the likelihood that early GBC may be missed in these patients. Likewise, while aggressive imaging in any patients who present with even the slightest suspicion might be helpful toward eliminating the barriers to early detection of GBC, the higher costs and side effects of frequent radiation exposure will lead many to question this approach. Our findings suggest that it may be useful to look for one or more of the paraneoplastic syndromes associated with GBC described in the section above. Because they can be easily diagnosed based on the results of a physical examination and routine blood work, physicians should consider GBC in the differential diagnosis should any paraneoplastic syndromes be detected, particularly in cases in which no other explanations emerge. This approach might be used to facilitate early diagnosis of many of the underlying carcinomas, including GBC.

## Conclusion

GBC is a rare disease typically diagnosed in its later stages after metastasizing to other organs. Early diagnosis may improve prognosis, as surgical removal of the entire gallbladder is easy to perform and generally associated with good postoperative results. Our manuscript describes several distinct paraneoplastic syndromes identified in patients that ultimately present with GBC. These syndromes typically have apparent signs and symptoms that might be used to recognize this frequently fatal neoplastic disease. Clinical knowledge of these syndromes and their association with GBC and other neoplastic diseases may be significant concerning early detection of the disease. While the existing evidence primarily relies on case reports, the presence of these syndromes should be evaluated in larger studies, such as case-control studies, before confirming them in more extensive cohorts.
